# Comparative *in vitro* activity of sulbactam with avibactam or durlobactam against carbapenem-resistant *Acinetobacter baumannii*

**DOI:** 10.1093/jacamr/dlaf098

**Published:** 2025-06-23

**Authors:** Ava J Dorazio, Ellen G Kline, Kevin M Squires, Marissa P Griffith, Yohei Doi, Ryan K Shields

**Affiliations:** Department of Medicine, Division of Infectious Diseases, University of Pittsburgh, Pittsburgh, PA, USA; Department of Medicine, Division of Infectious Diseases, University of Pittsburgh, Pittsburgh, PA, USA; Department of Medicine, Division of Infectious Diseases, University of Pittsburgh, Pittsburgh, PA, USA; Department of Medicine, Division of Infectious Diseases, University of Pittsburgh, Pittsburgh, PA, USA; Department of Medicine, Division of Infectious Diseases, University of Pittsburgh, Pittsburgh, PA, USA; Center for Innovative Antimicrobial Therapy, University of Pittsburgh, Pittsburgh, PA, USA; Departments of Microbiology and Infectious Diseases, Fujita Health University School of Medicine, Toyoake, Aichi, Japan; Department of Medicine, Division of Infectious Diseases, University of Pittsburgh, Pittsburgh, PA, USA; Center for Innovative Antimicrobial Therapy, University of Pittsburgh, Pittsburgh, PA, USA; Antibiotic Management Program, University of Pittsburgh Medical Center, Pittsburgh, PA, USA

## Abstract

**Objective:**

To determine the *in vitro* activity of sulbactam in combination with avibactam or durlobactam with and without meropenem or imipenem against carbapenem-resistant *Acinetobacter baumannii* clinical isolates.

**Methods:**

Standardized susceptibility testing by broth microdilution was performed to determine MICs for imipenem, meropenem and sulbactam alone, and for combinations including sulbactam/avibactam, sulbactam/durlobactam, sulbactam/avibactam/meropenem, sulbactam/avibactam/imipenem, sulbactam/durlobactacm/meropenem and sulbactam/durlobactam/imipenem. Whole-genome sequencing was also performed to compare MICs to key resistance determinants, including mutations in penicillin-binding proteins (PBPs).

**Results:**

Median sulbactam/durlobactam and sulbactam/avibactam MICs were 2 and 16 mg/L, respectively. Imipenem potentiated the *in vitro* activity of both combinations to a greater extent than meropenem corresponding to median sulbactam/durlobactam/imipenem and sulbactam/avibactam/imipenem MICs of 1 and 8 mg/L, respectively. Carbapenem combinations were more active than combinations without a carbapenem against isolates with PBP3 mutations.

**Conclusions:**

These data show that imipenem potentiates sulbactam-based combinations to a greater extent than meropenem; however, future studies are needed to define how these data should be applied in clinical practice.

## Introduction

Carbapenem-resistant *Acinetobacter baumannii* (CRAb) infections are a public health threat and remain atop the World Health Organization’s priority pathogens list.^1^ Historically, polymyxin-based combinations have been used for treatment, but two large randomized controlled trials demonstrated the unacceptably high mortality and toxicity associated with these regimens.^2,3^ Alternative options include high-dose ampicillin-sulbactam, tetracycline derivatives and cefiderocol; however, significant *in vitro* resistance, heteroresistance and pharmacokinetic/pharmacodynamic (PK/PD) concerns limit the utility of these agents,^4,5^ and clinical evidence supporting their broad application for CRAb infections is lacking. In pursuit of reliable treatment options, sulbactam has become the focal point for treatment given that it demonstrates intrinsic activity specific for *A. baumannii* through inhibition of penicillin-binding protein 3 (PBP3)^4^

Durlobactam is a new diazabicylooctane (DBO) β-lactamase inhibitor that inhibits class A, C and D β-lactamases, notably including OXA carbapenemases that are harboured by most CRAb. Indeed, the addition of DUR to SUL lowers median MICs by 32-fold against CRAb isolates.^6^ Among patients with CRAb pneumonia or bacteraemia randomized to receive sulbactam/durlobactam plus imipenem or colistin plus imipenem, rates of 28-day mortality were 19% (12/63) and 32% (20/62), respectively.^7^ Despite these encouraging results, a number of unanswered questions have been raised in extrapolating these data to real-world clinical practice. Foremost among them is the role of a carbapenem in combination with sulbactam/durlobactam. While imipenem was combined with sulbactam/durlobactam in clinical studies,^7^ meropenem is more frequently used in clinical practice and targets the same PBPs as imipenem.^8^ Next, sulbactam/durlobactam is currently only available in the USA, however, the burden of CRAb is generally greater in regions without access to the drug.^9^ Thus, it is important to understand whether alternative DBOs that are available in these regions can be combined with sulbactam, and what *in vitro* benefit adding a carbapenem may provide. The objectives of this study were to compare the *in vitro* activity of sulbactam/durlobactam versus sulbactam plus avibactam, and to determine whether imipenem or meropenem further potentiates the activity of either combination.

## Materials and methods

Fifty-eight clinical CRAb isolates collected from unique patients across five centres between 2017 and 2024 were included in the analysis;^5,10,11^ none had been previously treated with sulbactam/durlobactam. MICs of single agents, sulbactam, meropenem and imipenem were determined by broth microdilution in triplicate according to CLSI guidelines.^12^ Next, sulbactam was tested across a range of 0.12–128 mg/L with a fixed 4 mg/L concentration of avibactam or durlobactam. Finally, a 1:1 ratio of sulbactam/meropenem or sulbactam/imipenem ranging from 0.03 to 32 mg/L was also tested with fixed concentrations of avibactam or durlobactam as previously reported.^13^ Quality control strains *Pseudomonas aeruginosa* ATCC 27853, *Klebsiella quasipneumoniae* ATCC 700603 and *A. baumannii* ATCC 13 304 were tested with cefiderocol, meropenem and imipenem, and sulbactam-based combinations, respectively. Results were only reported when MICs were within expected ranges as available, including for sulbactam/durlobactam as a surrogate for other sulbactam-based combinations.^12^ Susceptibility to all sulbactam-based combinations was defined as an MIC ≤4 mg/L, the current sulbactam/durlobactam susceptibility breakpoint.^12^

Isolates underwent WGS on the Illumina platform; genome assembly and multilocus sequence typing were performed as described previously.^10,11^ ST was determined using the Oxford typing scheme.^14^ Antibiotic resistance genes were identified using ResFinder and AMRFinderPlus v.3.12.8.^15,16^ Unknown *Acinetobacter-*derived cephalosporinase (ADC) subtypes were manually confirmed. Protein sequences of *mrdA* (encoding PBP2) and *ftsI* (encoding PBP3) were compared with those of *A. baumannii* reference strain ATCC 17 978. All genomes are publicly available through NCBI (Table S1, available as Supplementary data at *JAC-AMR* Online). GraphPad Prism (version 10.2.3; Boston, MA, USA) was used for data visualization and continuous variable analysis via Mann–Whitney tests.

## Results

Across 58 isolates, 10 Oxford STs were identified (Table S1), including ST208 (*n* = 10), ST281 (*n* = 9) and ST451 (*n* = 20). All isolates harboured OXA-23- or OXA-40-like carbapenemase genes. Common ADC variants included ADC-30 (*n* = 20) and ADC-73 (*n* = 20). Mutations in PBP3 (encoded by *ftsI*) were identified in 53% (31/58) of isolates; including mutations A515V (*n* = 18), T511S (*n* = 1) and T526S (*n* = 1). Seven percent (4/58) of isolates harboured mutations in PBP2 (encoded by *mrdA*), all of which were P655A mutations. No isolate harboured metallo-β-lactamase genes (Table S1).

**Figure 1.**
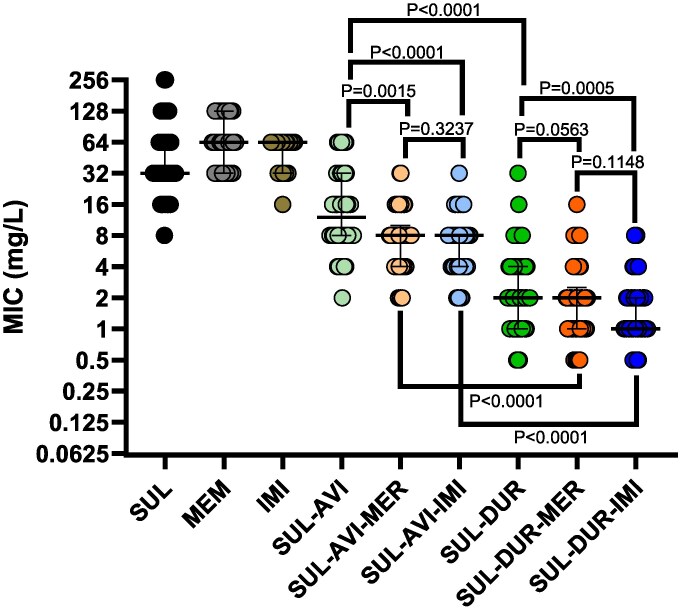
Comparison of sulbactam MICs in combination with avibactam or durlobactam with and without a carbapenem. Median MICs are denoted by a horizontal bar and interquartile ranges are shown by error bars. *P* values were calculated by Mann–Whitney tests comparing median MIC values between each column. Abbreviations. AVI = avibactam, DUR = durlobactam, IMI = imipenem, MEM = meropenem, SUL = sulbactam.

Median MICs for sulbactam, meropenem and imipenem were 32, 64 and >32 mg/L, respectively (Table 1). The median MIC fold reductions for sulbactam following the addition of avibactam or durlobactam were 4- and 16-fold, respectively. Overall, 19% of isolates demonstrated sulbactam/avibactam MICs ≤4 mg/L. Compared to sulbactam/avibactam, the median MIC fold-reduction for both sulbactam/avibactam/meropenem and sulbactam/avibactam/imipenem was 2-fold. The corresponding proportions of isolates with MICs ≤4 mg/L increased to 31% and 40%, respectively. By comparison, 83% of isolates demonstrated sulbactam/durlobactam MICs ≤4 mg/L. Corresponding proportions with MICs ≤4 mg/L for sulbactam/durlobactam/meropenem and sulbactam/durlobactam/imipenem were 88% and 95%, respectively. Compared to sulbactam/durlobactam, MICs were lowered by at least 2-fold for sulbactam/durlobactam/meropenem or sulbactam/durlobactam/imipenem in 41% and 60% of isolates, respectively. Across all sulbactam-based combinations, median MICs showed a stepwise decrease with sulbactam/avibactam being the least potent and sulbactam/durlobactam/imipenem the most potent (Figure 1).

**Table 1.** Summary of minimum inhibitory concentrations for sulbactam-based combinations against clinical carbapenem-resistant *A. baumannii* isolates (*n* = 58)

Agent (s)	MIC_50_ (mg/L)	MIC_90_ (mg/L)	MIC range (mg/L)	Percentage susceptible (%)^a^
MEM	64	>64	32–>64	0
IMI	>32	>32	16–>32	0
SUL	32	128	8–>128	0
SUL-AVI	16	>32	2–>32	19
SUL-AVI-MEM	8	16	2–32	31
SUL-AVI-IMI	8	16	2–32	40
SUL-DUR	2	8	0.5–32	83
SUL-DUR-MEM	2	8	0.5–16	88
SUL-DUR-IMI	1	4	0.5–8	95

AVI, avibactam, DUR, durlobactam, IMI, imipenem, MEM, meropenem, SUL, sulbactam.

Shading is added for readability.

^a^Percentage susceptibility was determined by CLSI interpretive criteria for meropenem, imipenem and cefiderocol. Susceptibility to sulbactam alone or in combination was defined as an MIC ≤4 mg/L.

**Figure 2.**
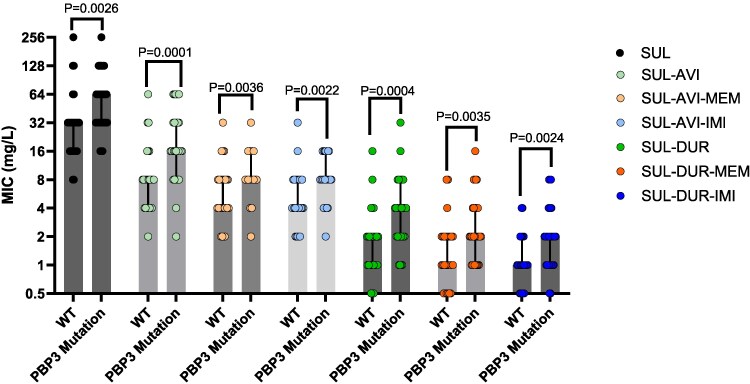
Sulbactam MICs alone and in combination against carbapenem-resistant *A. baumannii* clinical isolates with (*n* = 31) or without (*n* = 27) PBP3 mutations. Median MICs are denoted by a vertical bar for each group, and interquartile range are displayed by error bars. *P* values were calculated by Mann–Whitney tests comparing isolates with WT and mutated PBP3 for each group. Abbreviations. AVI = avibactam, DUR = durlobactam, IMI = imipenem, MEM = meropenem, SUL = sulbactam, WT = wild-type.

Median MICs were higher for SUL-based combinations against isolates with PBP3 mutations compared to wild-type (Figure 2). Against 31 isolates with PBP3 mutations, proportions with sulbactam/avibactam and sulbactam/durlobactam MICs ≤4 mg/L were 6.5% and 74%, respectively. Corresponding rates for sulbactam/avibactam/meropenem, sulbactam/avibactam/imipenem, sulbactam/durlobactam/meropenem and sulbactam/durlobactam/imipenem were 13%, 23%, 84% and 90%, respectively. Overall, 80% (8/10) of sulbactam/durlobactam non-susceptible isolates harboured PBP3 mutations; sulbactam/durlobactam/meropenem or sulbactam/durlobactam/imipenem demonstrated MICs ≤4 mg/L against 30% and 70% of these isolates, respectively. MICs did not vary by the presence of PBP2 mutations or by specific ADC variant.

## Discussion

The availability of sulbactam/durlobactam has provided optimism for effective treatment of CRAb infections; however, it is unclear how the agent should be best employed in real-world practice. Against a challenging set of CRAb isolates, we found that 17% of isolates were non-susceptible to sulbactam/durlobactam, a rate higher than previously reported in a global surveillance study,^6^ but in line with the rate reported against CRAb isolates non-susceptible to colistin and/or cefiderocol in the USA.^13^ In the latter study, a 3-drug combination of sulbactam/durlobactam/imipenem demonstrated MICs ≤4 mg/L against 97% of isolates, comparable to the rate of 95% reported here. For comparison, sulbactam/durlobactam/meropenem MICs were ≤4 mg/L against 88% of isolates, but did not lower sulbactam/durlobactam MICs against 59% of isolates. Thus, sulbactam/durlobactam/imipenem was the most active combination as demonstrated by the lowest MIC_50_ and MIC_90_ values (Table 1). Consistent with previous observations,^13,17^ the potentiation of sulbactam/durlobactam activity with either carbapenem was greatest among isolates with PBP3 mutations. Mechanistically, carbapenems exert their activity against CRAb through binding PBP2, which may offer complementary activity when sulbactam binding to PBP3 is compromised. Both meropenem and imipenem bind to PBP2 with high affinity,^8^ but the agents are differentially extruded by RND-type efflux pumps such as AdeABC and AdeIJK.^18^ Durlobactam itself may be a substrate for AdeIJK efflux systems,^19^ suggesting that further investigations are needed to explore the mechanistic rationale for differential carbapenem activity in combination with sulbactam/durlobactam. We hypothesize that imipenem is less likely to be affected by efflux-mediated resistance, and thus may be the preferred carbapenem to use in combination.

Durlobactam differs from AVI by a single double bond and additional methyl group resulting in more potent inhibition of class D OXA carbapenemases produced by CRAb.^20,21^ Unfortunately, DUR is not currently available in many regions affected by CRAb infections, thus prompting investigations into the potential of sulbactam/avibactam as an alternative.^22,23^ The underlying premise is that avibactam protects sulbactam from ADC-mediated hydrolysis and potentially some OXA-mediated hydrolysis resulting in a 2- to 4-fold MIC reduction compared to SUL alone.^22,23^ Our findings corroborate this, but underscore that the utility of this combination should be reserved for isolates that do not harbour PBP3 mutations given that sulbactam MICs are generally lower (Figure 2). Indeed, the median sulbactam/avibactam MICs were 16 and 8 mg/L for isolates with or without PBP3 mutations, respectively. Adding meropenem or imipenem to sulbactam/avibactam only modestly potentiated the activity of sulbactam/avibactam against PBP3 mutant isolates resulting in median MICs of 8 mg/L for both combinations. These data further confirm that durlobactam, and not avibactam, plays a more prominent role in protecting carbapenems from OXA-mediated hydrolysis, and importantly none of our isolates harboured metallo-β-lactamases. Moreover, it is clear from the literature that optimized sulbactam exposures are essential for treatment efficacy.^4^ Strategies to accomplish this may include administration of higher sulbactam doses and/or protection from β-lactamase hydrolysis. To this end, sulbactam/avibactam is clearly more potent than sulbactam alone *in vitro* (Figure 1). For regions without access to durlobactam, sulbactam/avibactam combination strategies merit further investigation. Importantly, like previous approaches to combine ceftazidime-avibactam plus aztreonam for metallo-β-lactamase-producing organisms,^24^ optimized dosing and co-administration practices will need to be defined.

In summary, these data add to the growing body of *in vitro* evidence to support adding a carbapenem to sulbactam/durlobactam, and provide new insights into the comparative potency of imipenem versus meropenem. Carbapenem potentiation was most discernible among isolates with PBP3 mutations given that sulbactam/durlobactam MICs are generally higher against such isolates. Clinical data are not yet available to know whether sulbactam/durlobactam monotherapy is sufficient to treat CRAb infections, or if efficacy is improved by adding a carbapenem. This study sheds new light on an alternative approach to combine sulbactam with avibactam. While sulbactam/avibactam-based combinations are not as potent *in vitro* as sulbactam/durlobactam-based combinations, avibactam can partially potentiate sulbactam activity. Clinical data have not yet been reported for sulbactam/avibactam against CRAb infections, and thus caution should be exercised. While the ideal approach to treat invasive CRAb infections remains elusive, the cumulative evidence is growing and can be used to define key research priorities moving forward.

## Supplementary Material

dlaf098_Supplementary_Data

## Transparency declarations

R.K.S. has served as a consultant for Allergan, Cidara, Entasis, GlaxoSmithKline, Melinta, Menarini, Merck, Pfizer, Shionogi, Utility and Venatorx, and has received investigator-initiated funding from Merck, Melinta, Roche, Shionogi and Venatorx. Y.D. has served as a consultant for Shionogi, GSK, Meiji Seika Pharma, Pfizer and AbbVie, has received honorarium from Shionogi, Gilead Sciences and BD, and has received investigator-initiated funding from Entasis. All other authors have no declarations.

## Supplementary data

Table S1 is available as Supplementary data at *JAC-AMR* Online.
